# Progress in Orthopædics

**Published:** 1900-12-29

**Authors:** 


### PROGRESS IN ORTHOPÆDICS.

**Spinal Curvature.**—Lovett [1] after studying the mechanics of scoliosis on the cadaver and by using models states that in side bending in the flexed position the bodies of the vertebræ rotate towards the convexity of the lateral curve, and that in the extended position the rotation is just the reverse. He concludes that

(1) Scoliosis must always be acquired in the flexed position.

(2) Reverse rotation is possible on the ground that the side flexion occurred in the extended position.

(3) Extension exercises should be cultivated in the treatment of lateral curvature.

(4) Symmetrical exercises are safer than complex unilateral ones.

(5) A safe and efficient treatment would consist in throwing the spine into such a position as would bring the articular processes into play and reverse the rotation acquired in the flexed position.

Noble Smith [2] lays stress upon the use of particular exercises in spinal curvature, holding that there can be no specific exercises laid down for all patients, but that each patient must have his exercises arranged for himself, since different people use different muscles in performing the same actions. He divides patients into four groups:—

Class I.—That in which the most notable condition is debility, occurring after illness or from some other cause, where the muscles are chiefly affected.

Class II.—Where laxity of the ligaments of the spinal joints are associated with laxity of the muscular system.

Class III.—Where alteration in the form of the bones has taken place.

Class IV.—In which the curves are more severe.

In the first three classes the conditions can be relieved by exercises; in Class IV., however, the effect will be less permanent, though good will undoubtedly be done.

He warns against the recommendation by some of exercises for the limb on the concave side of the curvature. These, he asserts, tend to increase the rotation of the vertebræ, the hardest feature to correct, since they use muscles — viz. trapezius, rhomboidei and latissimus dorsi—having for their attachment the spinous processes of the affected vertebræ. He suggests the use of these muscles on the convex side, and on the concave side of the curve the muscles which extend from the arm to the front of the body, in order to draw back the prominent side of the thorax.

Although, he continues, these exercises act upon the dorsal curve, yet the resistance of the pelvis to the action of the arms tends to twist the lumbar part of the spine in the opposite direction, and thus produces the exact action required.

The same authority [3] in treating on ambidexterity gives as causes for lateral curvature—standing on one leg; postures in drawing, writing, &c.; violin-playing, &c.; sitting in limp postures, when the predominating custom of using the right side more than the left causes the individual to rest with that arm protruded further forwards.

Jackson Clarke,[4] reviewing the subject of lateral curvature, flat-foot, knock-knee, &c., comments on the frequency of associated rickets and joint diseases, and he states that he believes, if carefully sought for at the time of the development of these deformities, rickets or arthritic tendencies will usually be forthcoming.

He further comments upon the association of pressure deformities with post-nasal vegetations. Nasal obstruction he finds frequently in cases of rickets, osteoarthritis, and gout.

Chisholm Williams [5] gave the periods of appearance of lateral spinal curvature from 500 consecutive cases in his experience, the chief period being from sixteen to twenty years.

From one to six years there were 35 cases; from six to ten years there were 66; from ten to sixteen years there were 121 cases; from sixteen to twenty years there were 175 cases; and over twenty years there were 105 cases. There were 131 cases of flat-foot among the lateral curvatures.

He believes that direct general muscular exercises do more harm than good, and directs his treatment to—

(1) The patient's general condition: improved by means of fresh air and food, fresh clothing allowing of chest development; medicinally by hypophosphites, cod-liver oil, and bland preparations of iron, &c.

(2) Getting the spinal column straighter by means of manual pressure, Barwell's rhachilysis, Barwell's lateral sling, and Volkman's sloping seat; weight pressure.

(3) Keeping up improved position by means of Chance's apparatus in use at the City Orthopædic Hospital, which allows muscular play, while supporting the spine and preventing any return to a worse position. It follows up the good results by being readjusted when the patient has become used to an improved position.

(4) Special exercises ordered for each weak muscle or group of muscles until the spine was straight, then general muscular exercises to assist in keeping it in position.

(5) Other aids, such as massage, galvanism, and rubber exercisers.

Tunstall Taylor [6] has devised an instrument he terms a kyphotone for stretching out a patient and so reducing an angular curvature as much as possible, before applying the plaster jacket. The device consists in an office-stool surmounted by a bicycle saddle. Means on either side are arranged for fixing the thighs and feet; an upright behind the stool carries rigid rods and handles and Sager's head-strap, with a pulley for extension. Behind the back is an adjustable rod with a small pressure plate "which can be adjusted to push forwards on the gibbosity from the posterior upright, and thus tend to make the kyphos the centre of the arc of lordosis, as is desired. With this instrument, it is held, a plaster jacket can be put on without assistance in a very short time, and fit very well, while fixing the spine in the most advantageous position for lessening the tendency for the production of deformity."

The same author [7] with McKim describes a "summer plaster jacket" for Pott's disease. A window is left over the abdomen, bony points of resistance and support being the pelvis, and sternum through the ribs. It is unnecessary to carry the plaster bandages more than an inch or more above the kyphos, which still further reduces the size of the jacket required. It is held to be quite as rigid and as good support as the old form.

Jones and Tubby [8] brought before the Clinical Society of London reports of 99 cases of manual rectification of angular curvature to substantiate their former propositions that (1) rectification of the deformity was not *per se* an immediate danger to life; (2) that the operation did not necessarily cause paraplegia; (3) by pressure many curvatures could be lessened and even obliterated. Their present conclusions were: (1) The danger to life was not great, without the use of much force; (2) the operation was a curative measure of paraplegia; (3) abscess formation was less frequent; (4) the risk of disseminating tubercle was a negligeable quantity; (5) there was no risk of a flail-like spine. The contra-indications were: (1) cervical and high dorsal curves unless associated with paralysis; (2) ankylosed spines; (3) large and angular deformities; (4) evidence of tubercle elsewhere; (5) in children under two and in adults over twenty-two; (6) in presence of abscess unless there were presence of paraplegia.

In the discussion following it was urged that the results after three years of wearing a mechanical appliance were just as favourable; and that the result of such treatment would only be apparent after further time had elapsed.

[1] Post. Grad., July. [2] Lancet, July 7. [3] Lancet, Sept. 1. [4] Brit. Med. Jour., Sept. 1. [5] Lancet, Sept. 1. [6] New York Med. Jour., May 12. [7] New York Med. Jour., May 26. [8] Brit. Med. Jour., March 31.

